# Reply: Asthma and cardiovascular disease: the strength of triangulation

**DOI:** 10.1183/13993003.00554-2024

**Published:** 2024-04-25

**Authors:** Chloe I. Bloom

**Affiliations:** National Heart and Lung Institute, Imperial College London, London, UK

## Abstract

In their correspondence, M.C. Tattersall and co-workers noted that our study reports contrasting findings to some earlier studies, including their own. They have raised several methodological points regarding our triangulation approach which leveraged two wholly different methods (traditional observational study and Mendelian randomisation) and multiple entirely different datasets [1]. We have discussed each of their concerns here.

## Shareable abstract

**A triangulation approach integrated different epidemiological methods and data sources (large observational study and Mendelian randomisation study) to provide more reliable findings and reveal potential sources of bias in previous asthma–CHD studies** https://bit.ly/3IXoK84

*Reply to M.C. Tattersall and co-workers:*

In their correspondence, M.C. Tattersall and co-workers noted that our study reports contrasting findings to some earlier studies, including their own. They have raised several methodological points regarding our triangulation approach which leveraged two wholly different methods (traditional observational study and Mendelian randomisation) and multiple entirely different datasets [1]. We have discussed each of their concerns here.

First, they comment that the observational study may have identified both type 1 and type 2 cardiac events, and that type 2 involves a secondary non-cardiac process. In our observational study, outcome events were only included if incident coronary heart disease (CHD) was the primary cause for the hospital admission. While a small proportion may have only been type 2 events, in a sensitivity analysis only including patients with a single cause for admission (indicating there was no additional process, such as sepsis or acute renal failure), we again found no increased risk. More importantly, in our Mendelian randomisation study – where we once more found no CHD risk associated with asthma – the outcome definition was obtained from the Coronary Artery Disease Genome-wide Replication and Meta-analysis consortium, and only included well-defined type 1 cardiac events.

Second, in our observational study we adjusted for healthcare behaviour to reduce the risk of detection bias (increased healthcare contact increases the chance of investigations for symptoms, even if mild). People diagnosed with asthma have an increased preponderance to attend their primary care practice for minor ailments, for example, a cold sore, tiredness or headache. M.C. Tattersall and co-workers commented that adjusting for this “may not have had the intended consequence” in relation to some patients having multiple comorbidities. We believe they mean we may have adjusted for other unmeasured comorbidities. Indeed, this was an additional intended benefit, as described in the methods, as these factors could also confound an association between asthma and CHD. We went on to demonstrate the problem of this confounding, as without such adjustment asthma falsely appeared to be associated with a significant increase in all-cause mortality. It is also notable that healthcare behaviour did not modify the association between asthma and CHD (*i.e.* there was no interaction), as there was no association with CHD found in patients with low GP attendance, nor in those with frequent GP attendance.

Third, they comment that we did not account for heterogeneity of asthma phenotypes and considered asthma as a homogeneous exposure. We find this comment surprising: in the observational study the patients were phenotyped by asthma disease severity, atopy and blood eosinophil counts. No association was found in any of the different asthma phenotypes.

Fourth, the authors express concern regarding horizontal pleiotropy in the MR study. However, our results remained consistent when we applied several methods robust to pleiotropy, and when we excluded possible pleiotropic single nucleotide polymorphisms (SNPs). In particular, they suggest that bias due to pleiotropy might be introduced by some of the SNPs being also associated with metabolic dysfunction, but we did not find evidence of this: none of the SNPs statistically identified as possibly pleiotropic were related to metabolic dysfunction. Moreover, if their hypothesis were true, one would expect to find an effect of asthma on CHD, not a lack of an effect as we found.

The causal link between COPD and cardiovascular disease is well established, with clear mechanistic hypotheses. In contrast, the postulated relationship between asthma and cardiovascular disease has been one of continual debate, with no clear hypothesis. Our study used robust methods and found no association with CHD, as well as demonstrating why previous observational studies may have been biased. Earlier studies have widely disagreed on which asthma patients are at risk of CHD: solely females, smokers, atopic or those with severe asthma [2]. This lack of consistency across studies further suggests possible study limitations, such as residual confounding, collider bias or reverse causality. We suggest a common reason for residual confounding is ignoring the use of corticosteroids, a widely recognised cause of cardiovascular disease [3, 4]. In some studies continuous corticosteroid exposure was used as the proxy for “persistent asthma”, which was reported to be the only asthma phenotype associated with systemic inflammation and predictors of cardiovascular disease (including carotid plaques) [5–7]. Such studies would have benefitted greatly from accounting for corticosteroid exposure, a major confounder in the postulated association between asthma and cardiovascular disease.

## Shareable PDF

10.1183/13993003.00554-2024.Shareable1 This one-page PDF can be shared freely online.
Shareable PDF ERJ-00554-2024.Shareable
