# Supplementary material for: Reply: Asthma and cardiovascular disease: the strength of triangulation
Source: Eur Respir J. 2024 Apr 25;63(4):2400554. doi: 10.1183/13993003.00554-2024 (PMC11043613; doi:10.1183/13993003.00554-2024)
Supplement: Supplementary file 1 [file ERJ-00554-2024.Shareable.pdf]

EUROPEAN RESPIRATORY JOURNAL
CORRESPONDENCE
C.I. BLOOM

SHAREABLE PDF

Reply: Asthma and cardiovascular disease: the strength of triangulation

Chloe I. Bloom

National Heart and Lung Institute, Imperial College London, London, UK.

Corresponding author: Chloe I. Bloom (chloe.bloom06@imperial.ac.uk)

Check for updates

Shareable abstract (@ERSpublications)
A triangulation approach integrated different epidemiological methods and data sources (large observational study and Mendelian randomisation study) to provide more reliable findings and reveal potential sources of bias in previous asthma–CHD studies https://bit.ly/3IXoK84

Cite this article as: Bloom CI. Reply: Asthma and cardiovascular disease: the strength of triangulation. *Eur Respir J* 2024; 63: 2400554 [DOI: 10.1183/13993003.00554-2024].

This extracted version can be shared freely online.

Copyright ©The authors 2024.

This version is distributed under the terms of the Creative Commons Attribution Licence 4.0.

Received: 19 March 2024
Accepted: 21 March 2024

Reply to M.C. Tattersall and co-workers:

In their correspondence, M.C. Tattersall and co-workers noted that our study reports contrasting findings to some earlier studies, including their own. They have raised several methodological points regarding our triangulation approach which leveraged two wholly different methods (traditional observational study and Mendelian randomisation) and multiple entirely different datasets [1]. We have discussed each of their concerns here.
